# Accuracy of continuous glucose monitoring during exercise-related hypoglycemia in individuals with type 1 diabetes

**DOI:** 10.3389/fendo.2024.1352829

**Published:** 2024-04-15

**Authors:** Kaisar Maytham, Per G. Hagelqvist, Susanne Engberg, Julie L. Forman, Ulrik Pedersen-Bjergaard, Filip K. Knop, Tina Vilsbøll, Andreas Andersen

**Affiliations:** ^1^ Clinical Research, Copenhagen University Hospital – Steno Diabetes Center Copenhagen, Herlev, Copenhagen, Denmark; ^2^ Center for Clinical Metabolic Research, Gentofte Hospital, University of Copenhagen, Hellerup, Denmark; ^3^ Section of Biostatistics, Department of Public Health, University of Copenhagen, Copenhagen, Denmark; ^4^ Department of Clinical Medicine, Faculty of Health and Medical Sciences, University of Copenhagen, Copenhagen, Denmark; ^5^ Department of Endocrinology and Nephrology, Nordsjællands Hospital Hillerød, University of Copenhagen, Hillerød, Denmark

**Keywords:** accuracy, continuous glucose monitoring, exercise, hypoglycemia, mean absolute relative difference, type 1 diabetes

## Abstract

**Background:**

Hypoglycemia is common in individuals with type 1 diabetes, especially during exercise. We investigated the accuracy of two different continuous glucose monitoring systems during exercise-related hypoglycemia in an experimental setting.

**Materials and methods:**

Fifteen individuals with type 1 diabetes participated in two separate euglycemic-hypoglycemic clamp days (Clamp-exercise and Clamp-rest) including five phases: 1) baseline euglycemia, 2) plasma glucose (PG) decline ± exercise, 3) 15-minute hypoglycemia ± exercise, 4) 45-minute hypoglycemia, and 5) recovery euglycemia. Interstitial PG levels were measured every five minutes, using Dexcom G6 (DG6) and FreeStyle Libre 1 (FSL1). Yellow Springs Instruments 2900 was used as PG reference method, enabling mean absolute relative difference (MARD) assessment for each phase and Clarke error grid analysis for each day.

**Results:**

Exercise had a negative effect on FSL1 accuracy in phase 2 and 3 compared to rest (ΔMARD = +5.3 percentage points [(95% CI): 1.6, 9.1] and +13.5 percentage points [6.4, 20.5], respectively). In contrast, exercise had a positive effect on DG6 accuracy during phase 2 and 4 compared to rest (ΔMARD = -6.2 percentage points [-11.2, -1.2] and -8.4 percentage points [-12.4, -4.3], respectively). Clarke error grid analysis showed a decrease in clinically acceptable treatment decisions during Clamp-exercise for FSL1 while a contrary increase was observed for DG6.

**Conclusion:**

Physical exercise had clinically relevant impact on the accuracy of the investigated continuous glucose monitoring systems and their ability to accurately detect hypoglycemia.

## 1 Introduction

Exogenous insulin replacement to obtain glycemic control is a hallmark for type 1 diabetes (T1D) treatment (1). The American Diabetes Association (ADA) recommends physical activity for individuals with type 1 diabetes as it improves glycemic control, decreases insulin requirements, and reduces cardiovascular complications in individuals with type 1 diabetes (2–5). However, exercise in these individuals is associated with hypoglycemia, which may be a barrier for obtaining glycemic control, and as a result many individuals with type 1 diabetes avoid engaging in regular physical activity (6, 7). Individuals with type 1 diabetes may need to consume considerable amounts of carbohydrates prior to physical activity in order to avoid exercise-induced hypoglycemia, which may reduce the potential benefits of vascular health and glycemic control that physical activity brings (8, 9). Continuous glucose monitoring (CGM) systems offer a way to frequently monitor glycemic changes throughout the day and particularly during exercise (10, 11). This provides individuals with type 1 diabetes a level of detail that cannot be achieved using capillary glucose meters, thus assisting in reduction of unnecessary glycemic fluctuations and episodes of hypoglycemia (12, 13).

Studies have indicated reduced accuracy of several CGM sensors during rapid glucose changes and low blood glucose levels, both commonly observed in individuals with type 1 diabetes and especially during exercise (14). CGM performance is clinically important since low sensor precision may lead to undetected events of hypoglycemia or unnecessary meal intake ensuing hyperglycemia. Here, we investigated the performance of two commonly used CGM systems, Dexcom G6 (DG6) and FreeStyle Libre 1 (FSL1), during plasma glucose (PG) decline and hypoglycemia, induced with or without exercise in individuals with type 1 diabetes.

## 2 Materials and methods

### 2.1 Approvals and registrations

CGM data presented in this study was obtained from a clinical trial investigating cardiovascular effects of exercise-related hypoglycemia in individuals with type 1 diabetes (registration with ClinicalTrials.gov, NCT04650646) (15). The trial was performed at Steno Diabetes Center Copenhagen and Center for Clinical Metabolic Research, Gentofte Hospital, University of Copenhagen, Hellerup, Denmark from September 2020 to June 2021. The study was conducted following the Helsinki Declaration and was approved by the Scientific Ethical Committee of the Capital Region of Denmark (ID No. H-20023688) and the Danish Data Protection Agency (ID No. P-2020-434). Written consent was obtained from all participants before being included in the study.

### 2.2 Study design

**Figure 1:**
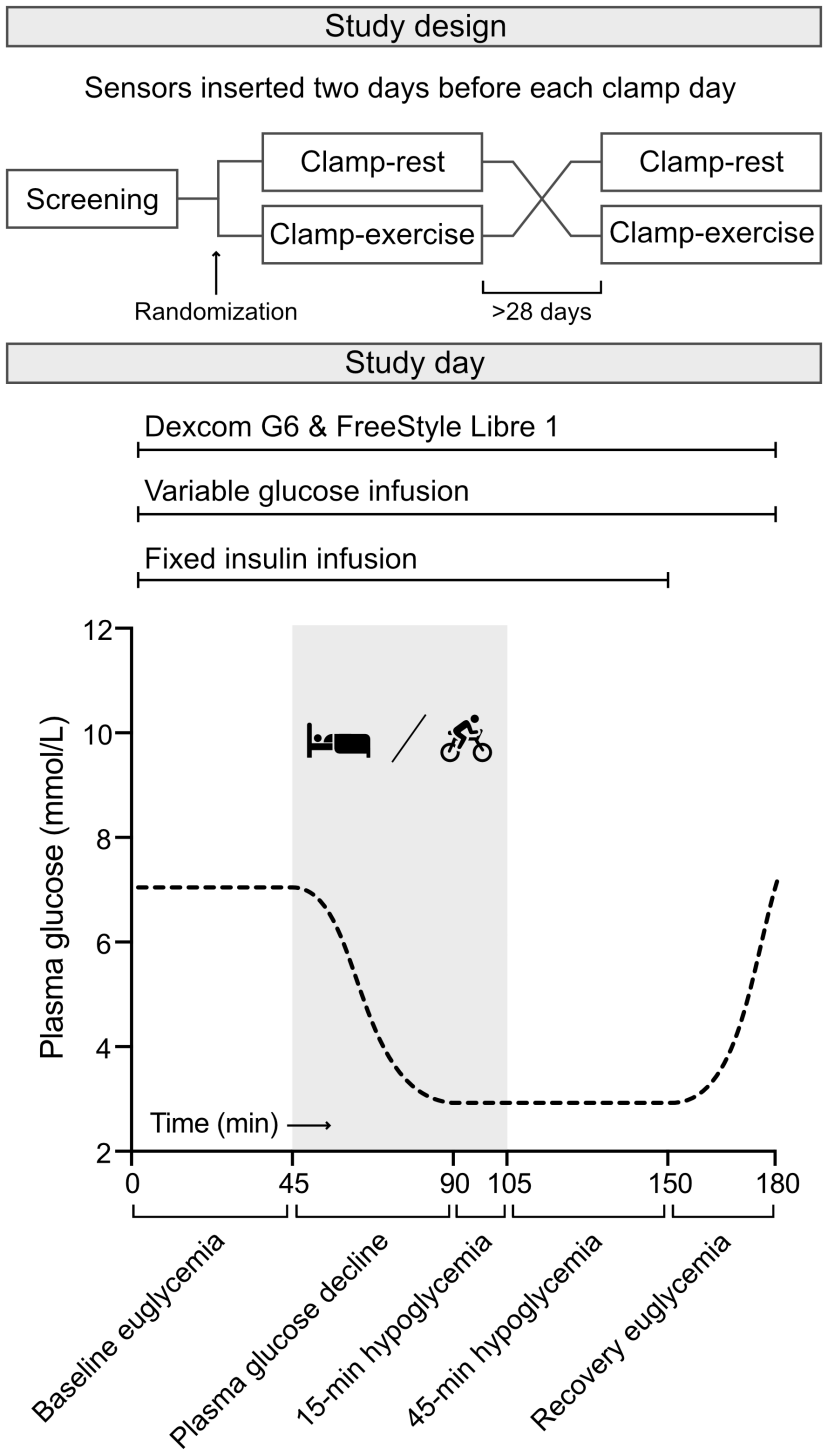
Study design. A randomized crossover euglycemic-hypoglycemic clamp study including Clamp-rest and Clamp-exercise. During Clamp-rest, participants were at bed rest during all phases. Clamp-exercise had an exercise element during plasma glucose decline phase and 15-minute hypoglycemic phase marked with gray (time 45 to 105 minute). Each clamp day was divided into five phases. Two continuous glucose monitoring systems, Dexcom G6 and FreeStyle Libre 1, were active throughout the clamp days. Further details and illustrations on the study design has been reported before (15).

Fifteen men diagnosed with type 1 diabetes participated in a randomized crossover study including two separate euglycemic-hypoglycemic clamp days. One clamp day included a bout of moderate-intensity cycling exercise performed during declining plasma glucose and hypoglycemia (Clamp-exercise). In the other clamp day, hypoglycemia was induced at rest (Clamp-rest). The participants were recruited from the outpatient clinic at Steno Diabetes Center Copenhagen, Herlev, Denmark. Data reported are a pre-planned secondary analysis from a previously published study. Hence, the sample size was calculated based of the primary aim of that study and has been reported elsewhere (15). The study design is illustrated in **Figure 1**. The two clamp days were separated by at least four weeks to rule out possible carry-over effects. Inclusion criteria were age ≥18 years, type 1 diabetes diagnosis according to World Health Organization (WHO) classification, C-peptide levels <200 pmol/L, insulin treatment for at least 1 year, and informed and written consent. Further details of inclusion and exclusion criteria have previously been reported (15).

### 2.3 Clamp procedure

Participants were supplied with and instructed to insert two CGM systems in parallel, Dexcom G6^®^ (Dexcom, Inc., San Diego, CA, USA) and FreeStyle Libre 1^®^ (Abbott Laboratories, Ltd., Alameda, CA, USA). The systems were inserted two days before the clamp days to reduce sensor inaccuracies (16). The participants were admitted in the morning after an overnight 10 hour fast including medicine fasting. Participants receiving insulin pen treatment were instructed to continue their usual basal insulin treatment, regardless of dosing time. Likewise, participants using insulin pump treatment were instructed to solely continue with the basal rate infusion throughout the test days. Sensors were not user-calibrated but relied on the factory calibrations as instructed in the devices’ user guide (17, 18). A peripheral intravenous catheter was inserted in the antecubital fossa of each forearm. One arm was heated throughout the clamp to obtain arterialized blood while the contralateral arm was used for isotonic saline (0.9% NaCl, Fresenius Kabi, Bad Homburg, Germany), insulin (Actrapid^®^; Novo Nordisk, Bagsværd, Denmark), and glucose (20% solution; Fresenius Kabi, Bad Homburg, Germany) infusion. The isotonic saline solution was administered at a constant infusion rate throughout the clamp to avoid volume depletion due to blood draw and to keep the intravenous cannula working properly. The hyperinsulinemic-euglycemic clamp was initiated at time 0 minutes when the target PG between 5.0 and 8.0 mmol/L was reached. A combination of a fixed insulin infusion rate at 80 mU/m2/min and a variable 200 mg/ml (20%) glucose infusion was initiated to clamp PG. Both clamp days (Clamp-exercise and Clamp-rest) contained the following phases: 1) a baseline euglycemic phase, 2) a PG decline phase induced at bed rest or during exercise, 3) a 15-minute hypoglycemic phase at bed rest or during exercise, continued by 4) a 45-minute hypoglycemic phase at bed rest and finally 5) recovery euglycemia. Exercise was performed at a moderate intensity defined as 64% to 76% of maximum heart rate calculated using the formula [207- (Age) × (0.7)] (19). The participants exercised on a Monark Ergomedic 839E (Monark Exercise AB, Vansbro, Sweden) for a total period of 60 minutes. Target heart rate was reached by adjusting the resistance of the cycle ergometer throughout the exercise period. The participants were instructed to begin exercise at a low-level intensity and gradually increase the intensity until reached target level. The target level of PG during the phases of hypoglycemia was <3.0 mmol/L, representing level 2 hypoglycemia (20). Glucose concentrations were determined every five minutes throughout the clamp days using DG6 and FSL1 in parallel. FSL1 was manually scanned during the clamp days whereas DG6 automatically stored the glucose values. Yellow Springs Instrument (YSI) 2900 biochemistry analyzer (Xylem, Inc., Rye Brook, NY, USA) was used to carry out the clamps as a PG reference method by sampling arterialized venous blood in 0.2 mL NaF tubes centrifuged at 7,400 g for 30 seconds and then analyzed.

### 2.4 Data and statistical analysis

For each sensor reading, the absolute relative difference was computed as the absolute difference between the reading and reference PG value divided by reference PG multiplied by 100 (21). For descriptive statistics, the absolute relative differences were summarized as mean ± standard error and plotted against clamp-time in figures. To compare the accuracy of each sensor between Clamp-exercise and Clamp-rest, we applied a linear mixed model with clamp time and clamp day and the interaction between them as fixed effect and with a heterogeneous compound symmetry covariance pattern to account for repeated measurements on each study participant. Results were reported as difference in mean absolute relative difference (ΔMARD) with 95% confidence interval for each clamp phase. Finally, a Clarke error grid analysis was performed for each clamp day to quantify the clinical significance of sensor inaccuracies (22). Paired sensor readings and reference PG values are depicted on a plot with five zones corresponding to varying clinical consequences. P <0.05 was considered statistically significant. SAS Studio version 3.8 (SAS Institute, Inc., Cary, NC, USA) was used to perform the linear mixed model analysis. The Clarke error grid plot was made with ega-package version 2.0.0 in R Statistical software version 4.2.1.

## 3 Results

**Figure 2:**
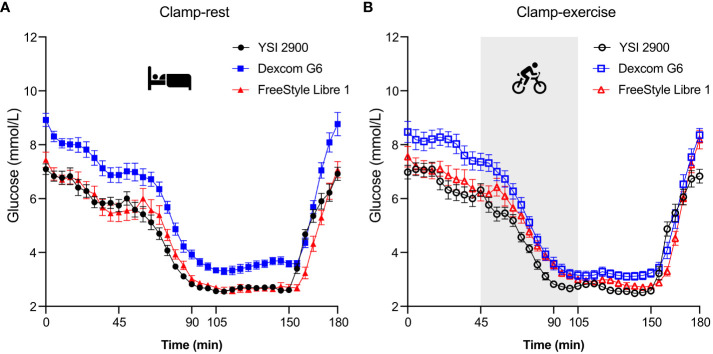
Glucose readings represented as mean ± SE. The clamp period totals 180 minutes and consists of 37 data points (data collected every five minutes). Clamp-rest **(A)** was at bed rest throughout the clamp period. Clamp-exercise **(B)** contained an exercise element on a cycle ergometer during plasma glucose decline phase and 15-minute hypoglycemic phase (time 45 to 105 minute). YSI, Yellow Springs Instrument.

All 15 participants (**Table 1**) completed both clamp days yielding 551 and 543 DG6 sensor-PG pairs for Clamp-rest and Clamp-exercise, respectively, as well as 491 and 512 FSL1 sensor-PG pairs for Clamp-rest and Clamp-exercise, respectively. All participants placed the sensors according to specified guidelines except one who placed FSL1 on the upper thigh. For both clamp days, mean PG was kept at 6-7 mmol/L during the baseline-euglycemic phase although slightly decreasing towards the decline phase (**Figure 2**). Target hypoglycemia was reached after 90-minutes and followed by steady-state hypoglycemia of <3.0 mmol/L. Overall, PG levels for both clamp days were comparable.

**Table 1:** Baseline characteristics of the study participants.

	Mean (SD) or N (%)
Males	15 (100%)
Age (years)	29.4 (8.1)
Body mass index (kg/m^2^)	23.7 (2.0)
Duration of type 1 diabetes (years)	13.1 (6.3)
HbA1c (mmol/mol)	51.0 (5.5)
HbA1c (%)	6.8 (0.5)
Fasting plasma glucose (mmol/L)	9.7 (2.1)
Heart rate (bpm)	64.8 (10.7)
Physical activity levels	
Low activity	3 (20%)
Moderate activity	3 (20%)
High activity	9 (60%)

Categorical data are presented as N (%), and continuous variables are presented as mean (SD). Further details on the participants have been reported before (15). bpm, beats per minute; HbA1c, glycated hemoglobin A1c; mmHg, millimeter of mercury; SD, standard deviation.

### 3.1 Comparison of accuracy between clamp days

**Figure 3:**
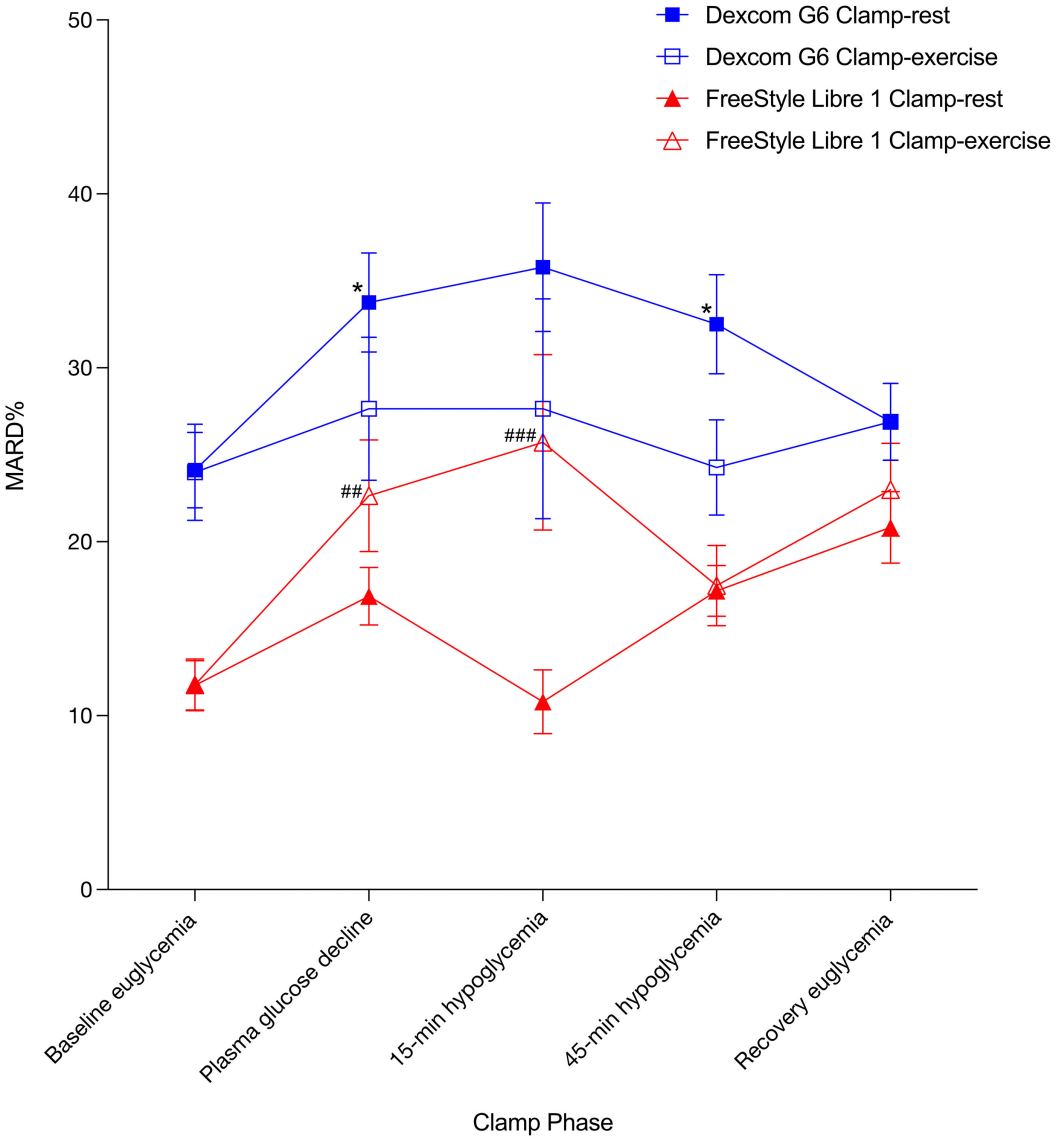
MARD% ± SE. * indicates significant difference between Dexcom G6 Clamp-exercise vs Dexcom G6 Clamp-rest. # indicates significant difference between FreeStyle Libre 1 Clamp-exercise vs FreeStyle Libre 1 Clamp-rest. Results at P < 0.05 was considered statistically significant and are shown with a single symbol. Results at P < 0.01 and P < 0.001 are shown with double and triple symbols, respectively. MARD%, mean absolute relative difference in percentages.

When comparing the clamp days (**Table 2**), FSL1 had a comparable MARD during phase 1 indicating similar baseline accuracies (**Figure 3**). Compared to rest, exercise increased MARD during phase 2 which continued during phase 3. There was a comparable MARD during phase 4 post exercise and during phase 5 recovery. DG6 had a comparable MARD between clamp days during phase 1, although this was surprisingly high compared to the expected (**Figure 3**). Compared to rest, exercise decreased MARD during phase 2 and phase 3, although not significantly. During phase 4, MARD was lower post-exercise, while a comparable MARD was observed between clamp days during phase 5.

**Table 2:** Comparison of Clamp-exercise and Clamp-rest throughout clamp phases.

Clamp phase	Sensor	ΔMARD%	Δ95% CI	*P*
Baseline euglycemia	DG6	-0.2	-4.3; 3.9	0.9227
	FSL1	0.0	-2.3; 2.4	0.9929
Plasma glucose decline (+/-exercise)	DG6	-6.2	-11.2; -1.2	0.0161
	FSL1	5.3	1.6; 9.1	0.0057
15-min hypoglycemia (+/- exercise)	DG6	-8.1	-16.3; 0.0	0.0505
	FSL1	13.5	6.4; 20.5	0.0005
45-min hypoglycemia	DG6	-8.4	-12.4; -4.3	<.0001
	FSL1	-0.5	-3.6; 2.6	0.7298
Recovery euglycemia	DG6	-2.8	-7.3; 1.6	0.2078
	FSL1	1.8	-3.0; 6.6	0.4680

Hypoglycemia was defined as plasma glucose <3.0 mmol/L, representing level 2 hypoglycemia. CI, confidence interval. ΔMARD%, mean absolute relative difference of Clamp-exercise – Clamp-rest in percentages.

### 3.2 Clarke error grid analysis of DG6 and FSL1

**Figure 4:**
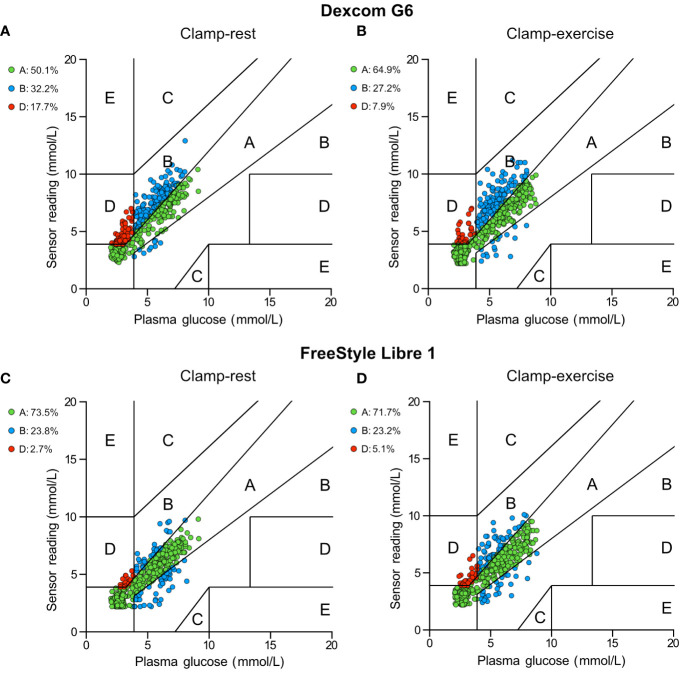
Clarke error grid analysis of Dexcom G6 and FreeStyle Libre 1. **(A)** Dexcom G6 for Clamp-rest. **(B)** Dexcom G6 for Clamp-exercise. **(C)** FreeStyle Libre 1 for Clamp-rest. **(D)** FreeStyle Libre 1 for Clamp-exercise. Each dot represents a glucose sensor reading paired with corresponding reference plasma glucose. Pairings in zone A and B are defined as clinically acceptable sensor estimates whereas pairings in zones C, D, and E are defined as clinically unacceptable and could lead to errors in diabetes treatment.

Assessing the clinical performance according to Clarke error grid analysis showed a difference between clamp days (**Figure 4**). FSL1 performance decreased during Clamp-exercise where data points in the combined zones A+B decreased compared to Clamp-rest and accordingly increased in the clinically unacceptable estimates zone D. DG6 performance increased during Clamp-exercise where data points in zones A+B increased indicating an increase in sensor estimates to more clinical acceptable accuracies. No data points were observed in zones C and E for all error grid analyses.

## 4 Discussion

We report that exercise can affect the sensor accuracy of the investigated CGM systems by either a decrease in sensor accuracy and the ability to detect hypoglycemia as seen with DG6, or contrary by an increase in sensor accuracy as seen for FSL1 which may be of clinical relevance for physically active individuals with type 1 diabetes when choosing between CGM systems.

The overall MARD of DG6 obtained in this study under baseline euglycemia was substantially higher compared to previous studies (23–25). Shah et al. compared DG6 sensor readings with YSI glucose values in 62 participants and demonstrated a general overall MARD of 9% (23). Generally, the design in the studies were similar e.g., utilizing a YSI analyzer, obtaining arterialized venous blood, and relying on the factory calibration. Furthermore, we followed the manufacturer’s specified guidelines for inserting and initializing the sensor and doing so two days before the clamp days to avoid possible inaccuracy during the sensor warm-up time. Since the overall FSL1 MARD of our study was more comparable with previous studies (26–29), and since our study followed the same principles as previous studies, the deviating MARD values obtained from DG6 may not be explained by the design of our study. The deviation may rather be related to the applied sensors. As the higher-than-expected MARD of DG6 was observed at baseline for both test days, and that they were comparable to each other, the comparison outcomes between clamp days were considered valid.

Our results suggest that exercise has a negative effect on FSL1 performance without affecting the post-exercise sensor performance. Contrary, DG6 had an improvement in performance during exercise which persisted post-exercise. We hypothesize that the exercise-related performance increase of DG6 is caused by increased blood circulation and production of heat during exercise resulting in a subsequent increase in skin blood flow (30). This could potentially lead to a higher interstitial space fluid turnover rate and better equilibrium between plasma and interstitial space fluid, thus more accurate estimates of glucose (31). Conversely, a worsening in performance was seen for FSL1 during exercise. This may be explained by the placement of the sensor in an area of high movement and mechanical activity during exercise as opposed to the abdomen as seen for DG6 (32). Thus, the continual mechanical movement of the sensor may have outweighed the possible performance increase from increased skin blood flow.

Few studies have assessed the sensors’ performance during exercise (33–37), although they mostly relied on capillary blood glucose as a reference method. Guillot et al. showed no notable changes in DG6 sensor accuracy during exercise while wearing the sensors on the abdomen (33). Dyess et al. showed an overall decrease in DG6 sensor performance during exercise, however apart from the abdomen, the participant had the option to wear the sensor on the upper arms, buttocks, and thighs (34). When sub-analyzing for the abdomen only, Dyess et al. found an increase in sensor performance which is in accordance with our study and supports our hypothesis about the role of placement of sensors.

For FSL1, Moser et al. showed higher MARD during exercise in 19 individuals with type 1 diabetes compared to capillary blood glucose (35). Likewise, Fokkert et al. showed an increase in MARD for FSL1 during exercise in 23 individuals with type 1 diabetes compared to capillary blood glucose (36). However, Giani et al. showed no performance difference between rest and exercise in 17 young individuals with type 1 diabetes (37). As FSL1 is placed on the upper arm, an area of high activity during exercise, the decrease in sensor performance may be of this reason. We can only speculate if the performance of FSL1 would remain unchanged or increase if placed on the abdomen during exercise compared to rest. However, Charleer et al. showed a worsening in FSL1 performance when placed on the abdomen (38). Although sensor placement may have a notable role, factors such as varying populations included, different exercise forms and intensities employed, different extent of glycemic excursions and varying PG reference methods utilized may contribute to differing results.

According to Clarke error grid analysis, most of the data points in our study were in the clinically safe zones A+B for both sensors regardless of the clamp day, although the percentage of data points in the upper zone D was surprisingly high for DG6 during Clamp-rest. During Clamp-exercise, DG6 had a shift in data points from the clinically unacceptable zones (solely located in the upper D zone) to the clinically acceptable zones with an increase in zones A+B by almost 10 percentage points compared to Clamp-rest. This indicates that exercise potentially improves sensor accuracy and/or sensor lag time during rapid PG decline and hypoglycemia. In contrast, FSL1 showed a slight decrease of data points in the clinically acceptable zones during Clamp-exercise compared to Clamp-rest. FSL1 had almost a doubling of data points in zone D for Clamp-exercise indicating exercise having a clinically relevant negative effect. Thus, Clarke error grid analysis is consistent with the MARD results and could be explained in the same manner.

The previously mentioned studies also evaluated the clinical safety of DG6 and FSL1 using Clarke Error grid analysis. Guillot et al. showed good clinical reliability for DG6 during exercise with 99% of all values located in the clinically safe zones A+B (33). Dyess et al. found that individuals who wore DG6 on their buttocks during exercise had an increase of values in zone D while an increase of values in zones A+B was seen when wearing the sensor on the abdomen (34). For FSL1, Giani found 97% of sensor readings fell in zones A+B during rest and 98% during exercise indicating a marginal difference between the two settings (37). Moser et al. found 91% of sensor values were in zones A+B during rest while 78% for FSL1 during exercise indicating a decrease in the clinical accuracy of the sensor during exercise (35). Throughout all the studies where exercise negatively impacted the sensor performance, sensor values specifically increased in the upper zone D similarly to our study indicating an increase in failure to detect hypoglycemia.

The strengths of the present study include the cross-over design, the direct comparison of exercise versus rest and the conduction in a controlled clinical research facility. Sensors were initialized two days prior to the clamp day to prevent possible sensor inaccuracies. Unlike other studies that used capillary blood glucose, our study utilized PG measured by YSI 2900 as a glucose reference method which is often cited as the gold standard (39). Furthermore, the sensors were not user-calibrated but relied on the factory calibration mimicking a real-life setting with individuals doing the same.

A limitation to our study is the small number of participants and that the study only included male adults thus limiting the generalizability to females and other age groups. One participant had their FSL1 placed on the upper thigh which could potentially influence on the results, although Charleer et al. showed a minimal difference between the placement of FSL1 on the upper thigh and the upper arm (38). Another limitation is the rather high MARD observed for Dexcom G6 which could influence the results and that MARD does not take sensor errors into account (e.g., consistent higher glucose estimates). To overcome this, an adjunctive analysis called precision absolute relative deviation, which requires the insertion of an identical parallel sensor, could potentially have added value to our study (40). A limitation of Clarke Error grid analysis is the rather stringent limits between the zones which newer error grids seek to mitigate (41). Finally, the physiological effect of physical activity on glucose levels is rather complex and different at differing exercise types, intensities, and durations. Thus, the observed performance of the sensors cannot be generalized to other exercise intensities or durations.

In conclusion, the two commonly used sensors DG6 and FSL1 showed different responses to exercise in relation to PG decline and hypoglycemia in individuals with type 1 diabetes. Exercise negatively impacted FSL1 sensor performance during both declining PG and hypoglycemia, whereas DG6 had more accurate sensor readings during exercise and post-exercise. Individuals with type 1 diabetes and healthcare practitioners should be aware of the potentially negative or positive impacts of exercise on CGM sensor accuracy in detecting clinically relevant episodes of hypoglycemia.

## Author's note

Parts of the following study were presented as an oral presentation at the European Association of the Study of Diabetes (EASD) 58th annual meeting in Stockholm, Sweden, September 2022

## Data availability statement

The raw data supporting the conclusions of this article will be made available by the authors, without undue reservation.

## Ethics statement

The studies involving humans were approved by the Scientific Ethical Committee of the Capital Region of Denmark. The studies were conducted in accordance with the local legislation and institutional requirements. The participants provided their written informed consent to participate in this study.

## Author contributions

KM: Data curation, Writing – review & editing, Writing – original draft, Visualization, Validation, Software, Project administration, Investigation, Formal analysis. PH: Writing – review & editing, Supervision, Resources, Methodology, Investigation, Funding acquisition, Data curation, Conceptualization. SE: Writing – review & editing, Validation, Resources, Methodology. JF: Formal analysis, Software, Supervision, Validation, Writing – review & editing. UP-b: Writing – review & editing, Methodology, Validation, Supervision, Conceptualization. FK: Conceptualization, Methodology, Resources, Supervision, Validation, Writing – review & editing. TV: Writing – review & editing, Validation, Supervision, Project administration, Funding acquisition, Conceptualization. AA: Conceptualization, Formal analysis, Methodology, Supervision, Validation, Writing – review & editing.
